# Nutrient resorption or accumulation of desert plants with contrasting sodium regulation strategies

**DOI:** 10.1038/s41598-017-17368-0

**Published:** 2017-12-06

**Authors:** Lilong Wang, Liang Wang, Wenliang He, Lizhe An, Shijian Xu

**Affiliations:** 1 MOE Key Laboratory of Cell Activities and Stress Adaptations, School of Life Sciences, Lanzhou University, Lanzhou, 730000 China; 2 Administration of Anxi Extra-arid Desert National Nature Reserve, Jiuquan, Gansu 736100 China

## Abstract

Desert plants are thought to rely more heavily on nutrient resorption due to the infertile soil. However, little is known regarding the phylogenetic effects on this traits, specifically for halophytes. Here we determined contents of nitrogen (N), phosphorus (P), potassium (K), sodium (Na), calcium (Ca) and magnesium (Mg) in 36 desert plants in a hyper-arid environment. The patterns of resorption or accumulation of the six elements were compared among plant groups with diverse leaf Na regulation strategies: i.e., euhalophytes (Eu), secretohalophytes (Se), pseudohalophytes (Ps) and glycophytes (Gl). Overall, N, P, K presented strict resorption across all groups, but no more efficient than global estimations. Ca and Mg tended to be resorbed less or accumulated during leaf senescence. Significant phylogenetic signal of both leaf Na content and plant group implies the pivotal role of Na regulation in the adaptation of plants to desert environment. Resorption proficiency, rather than resorption efficiency, is more phylogenetically conservative and more relevant to leaf functional traits.

## Introduction

In most terrestrial ecosystems, growth of wild plant is nutrient-limited^1^. Nutrient resorption, a process by which plant withdraws nutrients from senescing structures to developing tissues, is a critical mechanism to reduce the dependence on nutrient uptake and, consequently, increases plant fitness in low-nutrient environments^2^. Generally, nutrient resorption can be defined as resorption efficiency (RE), i.e., the proportion of green leaf nutrients withdrawn prior to senescence^3^ and as resorption proficiency (RP), i.e., the terminal nutrient concentration in senesced leaves^4^. Based on the global estimate, over 50% of leaf N and P (62.1%, 64.9% for N and P, respectively), varying among plant groups, are recycled via resorption^5^. As a critical trait in determining plant fitness, speculation about this process has suggested that plants in desert ecosystem may rely more heavily on the reabsorbed nutrients due to the poor soil fertility^6,7^. Comparing data from seven desert shrubs to non-desert shrubs was found the RE of N and P was higher in desert species^6^. Whereas, another study showed that shrubs in Chihuahuan desert were no more efficient or proficient at resorbing N and P than shrubs growing in other environments^8^. In part, the mixed results reflect the fact that drought may be an important factor in desert ecosystem affecting nutrient resorption^9^, as it will advance leaf senescence and, consequently, decrease nutrient resorption^10^. Although studies have noted lower N and P resorption at drier conditions^11,12^, controversial results have also been reported^13,14^. Such inconsistencies emphasize that more attention should be paid to resorption patterns of desert plants regarding the influence of drought and nutrient limitation.

Soil salinity is another common environmental problem in desert ecosystem due to the high surface evaporation^15^, which induce osmotic stress and ionic toxicity for plant growth^16^. Osmotic stress can advance leaf senescence, therefore, reduce nutrient resorption^9^. While ionic toxicity, primarily induced by Na, can reduce N uptake and disrupt whole-plant K homeostasis^16^, hinder nutrient resorption further. Although drought in conjunction with salinity poses the most severe environmental constraint to plant nutrient uptake and cycling^17^, it may also be proper conditions for halophytic species^18^. Desert halophytes are such remarkable plants which can not only tolerate salt concentrations, but also utilize Na as an osmotic solute in coping with water stress^16^. As they have evolved three tight leaf Na regulation strategies, i.e., compartmentation (euhalophyte, Eu, leaf- or stem-succulent species accumulating and sequestering salt within foliar tissues), secretion (secretohalophyte, Se, species with salt secreting glands) and rejection (pseudohalophyte, Ps, species limiting the entry of saline ions into transpiration stream)^19^. However, to the best of our knowledge, plants in desert saline environments have received limited attention in nutrient resorption, and the patterns of nutrient resorption in different halophytes remain unknown.

In addition to environmental factors, nutrient resorption is possibly associated with plant evolutionary history, an important intrinsic factor often neglected^4^. Without recognizing phylogeny, it is not possible to determine the adaptive significance of nutrient resorption^14^. Several studies have reported that more closely related taxa may have more similar resorption patterns of N and P than more distantly related taxa^4,14,20^. It should be noted that, those studies including phylogenetic effects are simple comparisons with no specific quantization standard, and often have been at broad taxonomic scales (i.e., angiosperms vs. gymnosperms)^20^ or at narrow taxonomic scales (i.e., congeners among several genera)^14^. While N and P are the main limiting nutrients globally, K, Ca, and Mg also play important roles in plant growth and function^21,22^. However, the influences of phylogeny on these essential cations are still unknown.

Here, by measuring six leaf element content in 36 species from 252 observations, we tested how the leaf resorption or accumulation patterns of five macronutrients (N, P, K, Ca, Mg) and Na differ in three groups of halophytes, as well as in glycophytes (Gl). Specifically, the two questions were addressed. (1) Are there different nutrient resorption patterns between four plants groups with contrasting Na regulation strategies? (2) Does phylogeny influence these patterns?

## Results

### Variations of chemical traits

**Figure 1:**
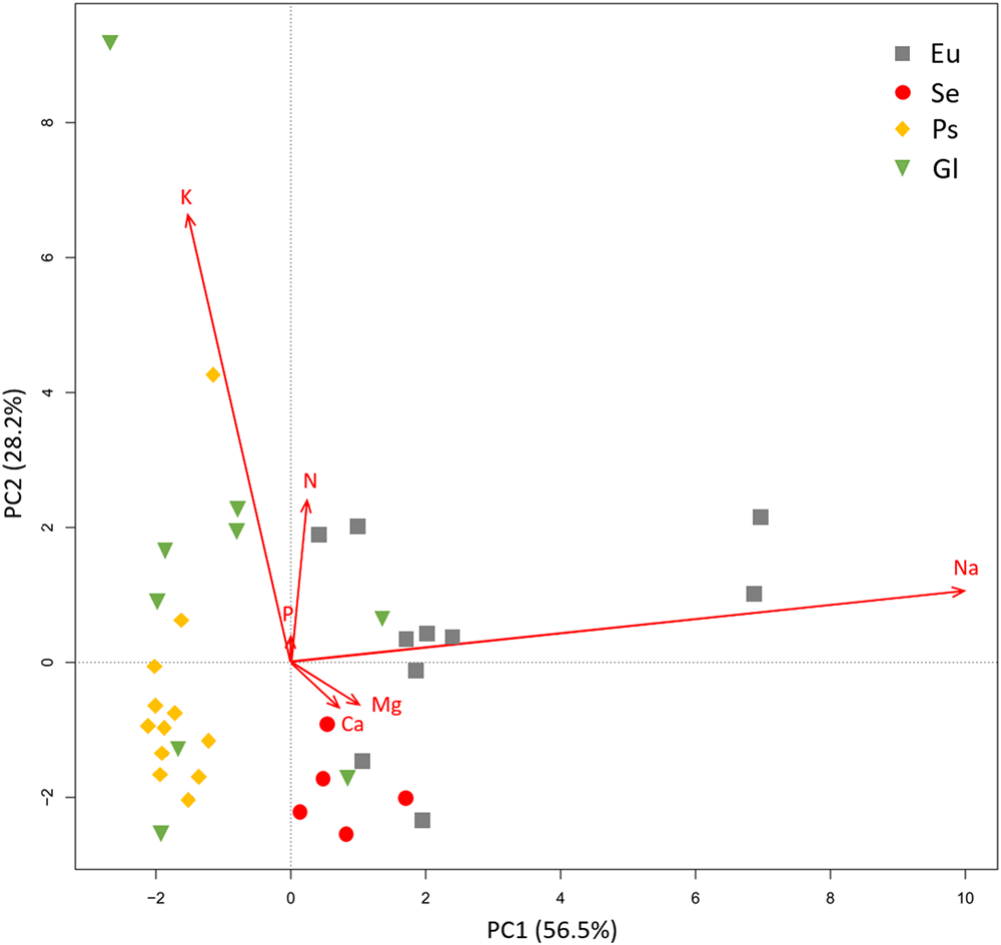
Principal component analysis (PCA) of the 6 leaf chemical traits. The ordination is based on 36 species and 6 elements.

**Figure 2:**
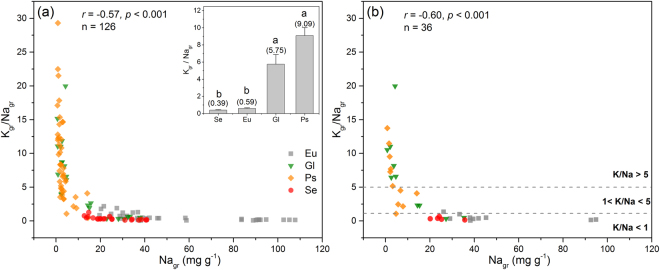
Correlations of green leaf potassium to sodium ratio (K/Na) and Na concentration. (**a**) Individual level, using all observations. The inset shows the average K/Na among plant groups, different letters above the error bars represent statistically significant differences (ANOVA, *P* < 0.05). (**b**) Species level, averaged by species.

Green leaves in Eu and Gl showed significantly higher N and P concentration than that in Se and Ps (Table 1). Se presented lowest leaf K concentration than the other three groups (Table 1). Both Ca and Mg concentration in leaves of Ps were significantly lower than that in Eu and Se (Table 1). Results of variance partitioning were dramatically different for N, P, K, Ca, Mg when employing family instead of plant group except for Na (Fig. S2). Plant group showed significant phylogenetic signal (K = 0.63, *P* = 0.002). The correlation structure for the six elements suggested that Na and K are more important in building the principal components (Fig. 1), however, there was no significant correlation between Na and K (*r* = 0.12, *P* = 0.49). Na concentration in green leaves was negatively correlated with K/Na ratio (Fig. 2). Significant phylogenetic signals of Na concentration and K/Na ratio were detected both in green and senesced leaves (Table 2).

**Table 1:** Element content in green and senesced leaves for different plant groups.

Element content (mg g^−1^)	Values (Means ± SE)			
	Eu (n = 10)	Se (n = 5)	Ps (n = 11)	Gl (n = 10)
Green leaf N	28.8 ± 1.1a	21.2 ± 0.6b	22.8 ± 0.7b	28.8 ± 2.2a
Green leaf P	2.84 ± 0.1a	2.09 ± 0.2b	2.15 ± 0.1b	3.31 ± 0.3a
Green leaf K	22.2 ± 1.9a	8.19 ± 0.8b	18.8 ± 1.6a	22.7 ± 3.6a
Green leaf Na	53.5 ± 4.7a	24.9 ± 2.0b	3.19 ± 0.5c	13.2 ± 2.8bc
Green leaf Ca	11.6 ± 0.7a	12.6 ± 0.8a	7.14 ± 0.7b	13.1 ± 1.8a
Green leaf Mg	19.0 ± 1.4a	23.9 ± 1.1a	12.5 ± 1.2b	11.6 ± 1.6b
Senesced leaf N	14.4 ± 0.8b	12.7 ± 0.5b	14.0 ± 0.9b	18.7 ± 1.4a
Senesced leaf P	1.40 ± 0.1b	1.02 ± 0.1c	1.25 ± 0.1bc	1.71 ± 0.1a
Senesced leaf K	12.6 ± 1.3ab	6.55 ± 0.7b	10.6 ± 1.3ab	13.3 ± 3.1a
Senesced leaf Na	70.2 ± 5.8a	20.3 ± 3.2b	4.54 ± 0.7c	20.0 ± 4.6bc
Senesced leaf Ca	12.4 ± 0.6b	12.1 ± 0.7b	9.43 ± 0.7b	17.5 ± 2.5a
Senesced leaf Mg	20.1 ± 1.4a	20.5 ± 1.0a	15.7 ± 1.4ab	13.8 ± 1.7b

Different letter represent significant differences at *P* < 0.05 level by one-way ANOVA. Values of each group are means averaged by species means. Species means are averaged by all individuals of each species.

**Table 2:** Phylogenetic signal of leaf chemical and functional traits.

		N	P	K	Na	Ca	Mg	N/P	K/Na	SLA	LDMC
Green	*K*	0.160	0.203	0.071	**0.498**	0.249	**0.459**	0.036	**0.642**	**0.414**	**0.815**
	*P*	0.277	0.196	0.766	**0.004**	0.066	**0.013**	0.872	**0.013**	**0.008**	**<0.001**
Senesced	*K*	**0.377**	**0.280**	0.195	**0.557**	**0.402**	**0.396**	0.216	**2.32**	—	—
	*P*	**<0.001**	**0.012**	0.426	**0.003**	**0.011**	**0.034**	0.070	**<0.001**	—	—
RE	*K*	0.166	0.052	0.171	0.243	0.140	0.208	—	—	—	—
	*P*	0.381	0.858	0.294	0.149	0.329	0.174	—	—	—	—

*P* < 0.05 (in bold) represent significant phylogenetic signal in the corresponding traits. The detailed description of K statistics see Blomberg *et al*. (2003).

### Characteristics of nutrient resorption

**Figure 3:**
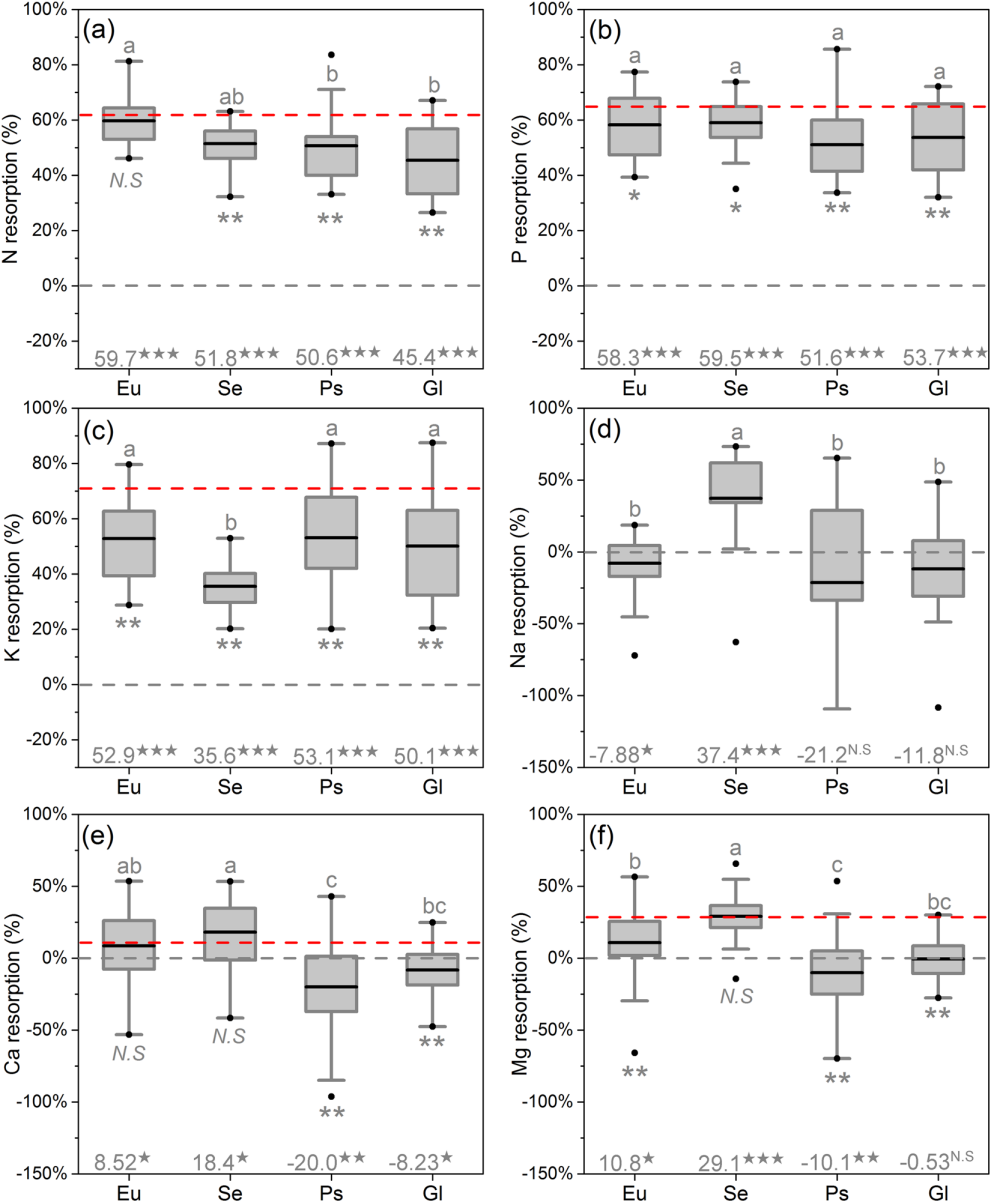
Boxplots of nitrogen (N), phosphorus (P), potassium (K), sodium (Na), calcium (Ca) and magnesium (Mg) resorption efficiency among plant groups (corrected for mass loss). The continuous line within each box shows the average, and error bars show 10th and 90th percentiles. Outliers are represented by small solid circles. Different letters above error bars represent significant different (ANOVA, *P* < 0.05). The gray dashed lines show 0% resorption. The red dashed lines represent global averages corrected with mass loss (Vergutz *et al*.^5^). Independent two sample *t*-test was used for statistical comparisons between this study and global averages, *N.S*, * and ** represent difference at *P* > 0.05, *P* < 0.05 and *P* < 0.01 level respectively. One sample *t*-test was used to assess the RE from 0% resorption efficiency. ★, ★★ and ★★★ represent difference at *P* < 0.05, *P* < 0.01 and *P* < 0.001 respectively.

**Figure 4:**
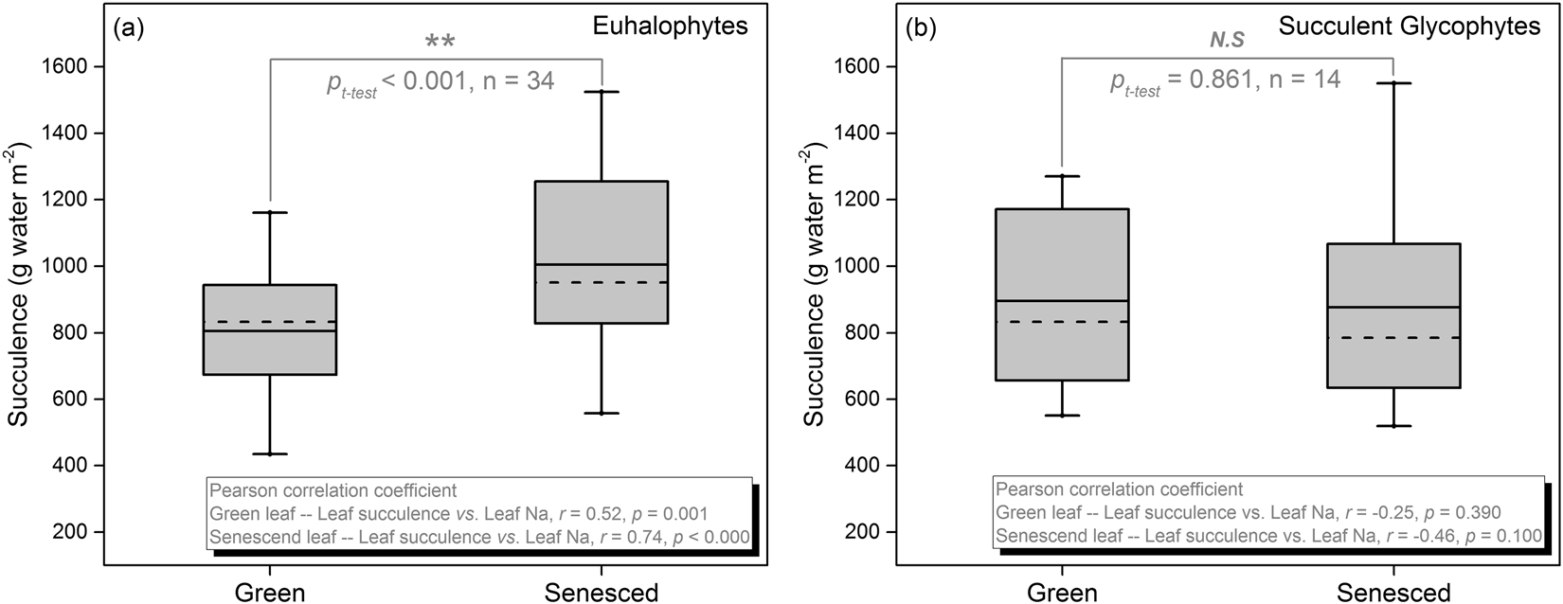
Succulence in green and senesced leaf of Euhalophytes (Eu) (**a**) and succulent glycophytes (**b**). The continuous line and dashed line within each box shows the average and median respectively, error bars show 10th and 90th percentiles. Independent two sample *t*-test was used to determine the succulence between green and senesced leaf, N.S represent no significant difference, ** represent significant difference at *P* < 0.001 level. The label within each graph shows the correlation coefficient of Pearson between succulence and sodium (Na) concentration both in green and senesced leaves.

Across the four plant groups, N, P, K presented strict resorption during leaf senescence, while Ca accumulations were found in Ps and Gl, and Mg in Ps (Fig. 3). Eu had higher NRE than Ps and Gl (Fig. 3). Gl showed the lowest NRP and PRP among the four plant groups (Table 1). The KRE in Se was significantly lower than that in the other three groups. In contrast, the KRP in Se was highest among the four groups (Table 1, Fig. 3). Positive correlations were found between NRE and PRE (Fig. S3), NRP and PRP (Fig. S4), as well as CaRE and MgRE (Fig. S3), respectively. Significant phylogenetic signal was more commonly detected in RP rather than in RE (Table 2). Only Eu showed significant accumulation of Na (−7.88%) during leaf senescence (Fig. 3). Significant positive correlations between leaf succulence and Na concentration was detected in Eu rather than in succulent Gl (Fig. 4).

### Relationships between leaf functional traits and nutrient resorption

At individual level, the RPs of N, P and K were negatively correlated with specific leaf area (SLA) and were positively correlated with leaf dry matter content (LDMC) (Table 3), respectively. More significant correlation was found between RE and LDMC rather than between RE and SLA (Table 3). At species level, neither SLA nor LDMC was correlated with RE, while significant correlations were detected between LDMC and the RP of the other five elements except for N (Table 3).

**Table 3:** Covariations between leaf functional traits (SLA, LDMC) and resorption efficiency (RE) or resorption proficiency (RP).

Traits-Nu Re	Individual (n = 123)		Species (n = 36)		Species PIC (n = 35)	
	*r*	*P*	*r*	*P*	*r*	*P*
SLA-NRE	−0.02	0.836	−0.08	0.636	0.22	0.206
SLA-NRP	−**0.29**	**<0.01**	−**0.38**	**0.021**	−0.30	0.082
SLA-PRE	0.01	0.895	0.15	0.383	0.03	0.848
SLA-PRP	−**0.28**	**<0.01**	−**0.33**	**0.047**	−0.30	0.076
SLA-KRE	0.16	0.076	0.13	0.454	0.23	0.190
SLA-KRP	−**0.19**	**0.039**	−0.30	0.080	−0.17	0.335
SLA-NaRE	−0.12	0.171	−0.28	0.093	0.07	0.691
SLA-NaRP	**0.31**	**<0.01**	0.26	0.130	0.11	0.513
SLA-CaRE	−0.16	0.079	−0.11	0.525	0.20	0.249
SLA-CaRP	**0.28**	**<0.01**	0.23	−0.177	0.20	0.244
SLA-MgRE	−0.22	0.016	−0.12	0.477	0.08	0.663
SLA-MgRP	0.01	0.956	−0.01	0.971	0.02	0.926
LDMC-NRE	−**0.31**	**<0.01**	−0.23	0.170	−**0.48**	**<0.01**
LDMC-NRP	**0.24**	**<0.01**	0.28	0.100	−0.01	0.970
LDMC-PRE	−**0.26**	**<0.01**	−0.31	0.062	**0.53**	**<0.01**
LDMC-PRP	**0.32**	**<0.01**	**0.37**	**0.026**	**0.35**	**0.037**
LDMC-KRE	−**0.24**	**<0.01**	−0.19	0.281	−**0.44**	**<0.01**
LDMC-KRP	**0.37**	**<0.01**	**0.44**	**<0.01**	**0.64**	**<0.01**
LDMC-NaRE	**0.26**	**<0.01**	0.24	0.168	0.26	0.128
LDMC-NaRP	**0.53**	**<0.01**	**0.48**	**<0.01**	**0.34**	**0.043**
LDMC-CaRE	−0.09	0.318	−0.01	0.974	0.07	0.697
LDM-CaRP	**0.32**	**<0.01**	**0.35**	**0.034**	**0.35**	**0.040**
LDMC-MgRE	−0.08	0.396	−0.03	0.851	−0.27	0.116
LDMC-MgRP	**0.31**	**<0.01**	**0.37**	**0.027**	−0.10	0.559

SLA, specific leaf area. LDMC, leaf dry matter content. The RE was corrected with mass loss. Significant relationships (*P* < 0.05) are presented in bold. PIC phylogenetically independent contrast.

## Discussion

Based on the measurement of six element contents (N, P, K, Na, Ca, Mg) both in green and senesced leaves of halophytes and glycophytes in an extra-arid environment, the patterns of resorption or accumulation of these elements were explored. The diverse leaf Na regulation strategies are phylogenetically conservative, implying the pivotal role of Na in the survival of plants in desert environment. Over 50% of N and P were resorbed during leaf senescence, indicating nutrient resorption is one of the key component of plant nutrient conservation strategies in water-stressed environment. Significant Na accumulation with leaf senescence is ubiquitous in groups of Eu. Compared with RE, RP is more phylogenetically conservative and more relevant to leaf functional traits (SLA, LDMC).

### The divergent Na regulation implies its role in the survival of different plant groups

Across all sampled species in the study are, over 50% of leaf Na variation was explained by plant group (Fig S2a). The higher Na concentration in leaves of Eu, rather than in Se and Ps, is primarily owing to its high degree of leaf succulence, a special feature to take advantage of the Na in coping with salt stress^16^. Since substantial amount of Na is compartmentalized in the vacuole of Eu, organic osmolytes, such as amino acids, non-protein amino acids, quaternary ammonium compounds and polyamines, are needed in the cytoplasm to prevent adverse effects on metabolism^23,24^. The lowest Na concentration in Ps may be attributed to the fact that Ps can prevent Na uptake by roots^25^. Therefore, Ps is a type of “salt-avoiders” that actively escapes from salinity by locating the active part of the root system into deeper soil^26^. Interestingly, Na may be a “cheap” osmotic solute for some glycophytes^27,28^. For instance, *Zygophyllum xanthoxylum*, a succulent Gl, accumulates large amount of Na in leaf but only adapts to gravel desert across the study area. In this kind plant, Na should also perform function of osmotic regulation instead of K under low NaCl treatment^29,30^. However, the replacement of the non-osmotic functions of K by Na may not be achieved both for glycophytes and halophytes^27^, hence, the ability to accumulate essential nutrients particularly K is vital for plants adaptation in saline environment^16^. Across the four plant groups, despite of the large difference in Na concentration, no significant difference was found in K concentration among Eu, Ps and Gl (Table 1), which also leads to contrasting leaf K/Na ratios among these functional groups. It appears, when K/Na ratio >5, the contribution of K to the osmotic balance is larger than Na because the corresponding Na concentration in green leaf was strictly below 5 mg g^−1^, which is significantly lower than the mean values (8.91 mg g^−1^) of terrestrial plants across China^31^. In contrast, when K/Na ratio <1, it allows plants to use Na as the major osmoregulatory substance^16,28^.

### Resorption of N, P and K may not be more efficient in desert plants

Across the four plant groups, over 50% of N and P are recycled via resorption, which reflects that nutrient resorption is a critical nutrient conservation strategy especially for desert plants^32^. It seems reasonable to expect desert plants to rely more heavily on nutrient resorption, because desert environments afford plants less of a chance to recuperate nutrients lost in abscised litter due to the drought-induced slow decomposition^6^. However, compared to global averages, our results indicate significantly lower RE for N, P and K both in halophytes and glycophytes (Fig. 3). In addition, the RP of N and P are well above the incomplete range^4^ for most of the species (Fig. S3), which suggests that, although in nutrients poor environment, both of halophytes and glycophytes are neither more efficient nor more proficient in nutrient resorption, especially for N and P. This may be attributed to the fact that drought and salinity are the primary factors constraining nutrient uptake and resorption in desert environments^10^. In addition, the leaf economics spectrum demonstrates that species adapted to stress environments tend to employ “slow-return” strategies, typically with lower SLA and lower growth rates^33^. In this work, the SLA of the 36 species located in the lower end of terrestrial plants^34^ and negatively correlated with the RP of N, P and K (Table 3). Therefore, the low SLA may not only be an adaptive strategy for desert plants in coping with drought, but also be a beneficial trait for them to be more proficient in nutrient resorption under nutrient-poor conditions. The higher NRE in Eu (Fig. 3) but the similar NRP among Eu, Ps and Se (Table 1) demonstrates that Eu should be halophyte with more efficient but less proficient in N resorption. This may be caused by the accumulation of non-structural N compounds in Eu leaves, which precludes more proficient in N resorption^35^. As for K, our data suggest that the extra low K content in green leaf (Table 1) may limit K resorption in Se.

### Resorption or accumulation of Na, Ca, and Mg during leaf senescence

As the two most important structure elements in cell walls and chlorophyll molecule, Ca and Mg tend to be resorbed less or accumulated (Fig. 3). This is consistent with previous study, and can be due to the traits of low phloem mobility and often enriched during leaf senescence^36,37^. Na is very similar to K in the active mobility and physic-chemical properties^27^. Therefore, various opinion on resorption or accumulation of Na has been raised for long time. In the present study, significant Na accumulation during leaf senescence of Eu (Fig. 3) is positively correlated with the significant leaf succulence development (Fig. 4a). In contrast, neither Na accumulation nor correlation between Na concentration and leaf succulence was detected in succulent Gl (Fig. 4b). These results support the idea that succulence development is a consequence of Na accumulation in older leaves of succulent halophytes^36^. Generally, Na is harmful for plants because of its toxicity^19^. However, our data indicated a substantial “resorption” of Na in leaves of Se during leaf senescence. Considering the desalination effects, that means excess salt ions can be secreted out of leaf surface by salt glands^16^, the calculation method for RE may not apply to Na in leaves of Se. Another experiment should be designed and more evidence should be employed to discover the reason of reduction of Na in senescenced leaves, resorption or secretion, or both. Ps prefers preventing Na uptake by roots rather than utilizing Na as a “cheap” osmotic solute^25^. Therefore, Ps is able to maintain leaf Na concentration well below the toxic level and the resorption or accumulation of Na may be useless for Ps in coping with salt stress^26^. In summary, Na accumulation in fallen leaves may be a specific adaptive strategy for Eu to avoid ionic toxicity.

### Phylogeny acts upon RP, not RE

In the context of plant evolution, adaptive variation has been addressed two complementary perspectives, divergent versus convergent evolution^38^. Previous studies suggested that RP of N and P appeared to parallel some phylogenic trends, that means the similar resorption patterns generally are found in closely related taxa not in weakly related taxa^4,14^. Across all species in this study, significant phylogenetic signals were detected in RP for five elements, except for K. In contrast, there were non-significant phylogenetic signals in RE for the total six elements. Therefore, phylogeny does exert influence over RP rather over RE^39^, thus RP may be more phylogenetically conservative. Since the RP alone may mask the realized degree to which plants can conserve nutrients invested in foliage, especially in water-stressed environments^35^, RE in concert with RP may be most advantageous to the understanding of the resorption process. Variance partitioning of leaf Na concentration indicated that the variance components of plant group are very close to that of family (Fig. S2), besides, plant group also showed significant phylogenetic signals (K = 0.63, *P* = 0.002). These results suggest that more closely related taxa tend to employ similar Na regulation strategies. It also seems likely that the distribution of Na regulation strategies is not random over phylogeny.

## Conclusions

In summary, we explored contents of five macroelements and Na both in green and senesced leaves of desert plants with diverse Na regulation strategies in a hyper-arid desert in northwest China. N, P and K showed strict resorption across all plant groups, but no more efficient than global estimations, which suggests that water stress may be the primary factor constraining plant nutrient uptake and resorption in desert environments. Ca and Mg tend to be resorbed less or accumulated during leaf senescence. The significant phylogenetic signals of both leaf Na content and plant group implies the tight Na regulation strategies and its pivotal role in the adaptation of plants to drought and salinity. Compared with RE, RP is more phylogenetically conservative and more relevant to leaf functional traits (SLA, LDMC). In desert ecosystems, it should be more advantageous to understand the resorption process by RE in concert with RP.

## Materials and Methods

### Site description

Research took place in the Anxi Extra-arid Desert National Reserve (39°52′–41°53′N, 94°45′–97°00′E), west end of Hexi Corridor in Guazhou County, Gansu province, China. The reserve represents a hyper-arid desert ecosystem (aridity index <0.02), with mean annual temperature of 8.7 °C, mean annual precipitation of 45 mm and annul evaporation of 3000 mm^40^. Most of the reserve is typical gravel desert containing large amount of gravel and extra-low moisture content, but with non-saline soils. The Shule River, an interior river runs through the lower areas of the reserve and results in soil salinity due to the high surface evaporation. The saline soils are sandy clay with relatively higher moisture content (Table S1). The vegetation is dominated by glycophytes in gravel desert, and by halophytes in saline area, respectively (Table S1).

### Field survey and sampling

A 20 × 20 m plot was randomly established at each of 18 sites (13 sites in saline environments, 5 sites in gravel desert). During the peak growing period (middle of July 2015), sun-exposed and fully expanded green leaves were collected from 5 to eight healthy individual for each woody species and then marked with metal tag. Three 1 × 1 m tagged subplots were applied for sampling of herb species in each plot. At the end of growing season (early October 2015), recently senesced (often yellow), but still attached leaves were collected from the same tagged individual or subplot by gently flicking the branch or leaf. The sampled leaves were rinsed with deionized water to remove surface salts and dust by using a spray bottle in the field. For each species and each sample, at least 60 g of fresh leaves (mixed uniformly with individual) were collected, of which about 10 g were stored in ice box to keep fresh and the rest were stored in paper envelopes for chemical analyses. Triplicate soil samples were randomly taken by an auger from different layers (0–20, 20–40, 40–60 cm) within the plot, where each replicate comprised a mixture of three adjacent cores.

Overall, 252 leaf samples of 36 species belonging to 15 families were collected within the study area. Plants were divided into four groups, including glycophytes (Gl), i.e., non-halophytic plants growth in gravel soils which do not exist in saline environments, and three groups of halophytes, i.e., Eu, Ps and Se. The division of the four plant groups was according to *Flora in Desertis Reipublicae Populorum Sinarum* ^41^ and the latest edition of *Halophytes in China* ^42^.

### Trait measurement

The leaf dry matter content (LDMC) was obtained by the ratio of leaf saturated weight/leaf dry weight^43^. The specific leaf area (SLA, ratio of leaf area/leaf dry weight) was measured by a photographic method^43^ and analyzed using ImageJ (National Institutes of Health, USA, http://imagej.nih.gov/ij). Leaf succulence was obtained by the ratio of leaf water content/leaf area^36^, values larger than 500 indicate significant succulence. Leaf samples stored in paper envelopes were oven dried at 60 420 °C to a constant weight, and ground into fine powder using a ball mill (MM200, Retsch, Haan, Germany) to enable chemical analysis.

Air-dried soil samples were sieved (2 mm), and samples for total N and P were further pulverized through 100-mesh sieve (0.15 mm). Soil pH and electrical conductivity (EC) were measured on 1:2.5 and 1:5 soil: water extracts respectively. Total N in soils (0.15 mm sieved) and leaves (fine powder) were measured by an elemental analyzer (FLASHEA 1112 Series CNS Analyzer, Thermo, USA) and total P were measured using the ammonium molybdate method after persulfate oxidation^34^.

Before the analysis of cations, leaf samples (fine powder) were digested in the mixed acid (HNO_3_:HClO_4_:H_2_SO_4_, 8:1:1, volume ratio) in 420 °C. Soil samples were measured on 1:10 soil:water extracts (soil soluble cations). The atomic absorption spectroscopy (iCETM 3300, Thermo, USA) was used to determine the concentration of Na, K, Ca, Mg in leaf digestive production and soil extracts.

### Nutrient resorption calculations

The resorption proficiency (RP) was defined as the nutrient content in senesced leaves, higher resorption proficiency is corresponding to lower final nutrient concentrations in senesced leaves^4^. The Resorption efficiency (RE) was quantified as the proportional withdrawal of a nutrient or element during senescence^44^ and expressed as Eq. (1):

1 $$RE=\frac{N{u}_{green}-N{u}_{senesced}}{N{u}_{senesced}}\times 100 \% $$

where Nu_green_ and Nu_senesced_ are mass based nutrient concentrations in green and senesced leaves.

Considering the leaf mass loss during senescene, the mass loss correction factor (MLCF) could be used to compensate the underestimation of RE^45^. In this study, the MLCF was calculated as the ratio of dry mass of senesced leaves and the dry mass of green leaves^5^. The RE for analysis were corrected using Eq. (2):

2 $$RE=(1-\frac{N{u}_{senesced}}{N{u}_{green}}\times MLCF)\times 100 \% $$

### Statistical analysis

To account for the variation in sample size for each species, data were analyzed at two levels i.e., species level, using species means, individual level, using all data. Differences between plant groups were tested using one-way ANOA while the “Independent two-sample *t*-test” was used to test the differences of RE between data obtained in this study and in global scale. Principal component analysis (PCA) was used to evaluate the correlation structure of the six elements in plant leaf, and was performed in the “vegan” package^46^. Bivariate relationships were examined with standardized major axis regression (SMA) as there are no independent or dependent variables and measurement error exists for both variables, and was performed in “smatr” package^47^. General linear model (GLM) was used to quantify the contribution of soil (different sites) and plant group/ family to the total variance of leaf chemical traits.

Based on Angiosperm Phylogeny Group III (APG III) classification of angiosperms^48^, the phylogenetic tree of all species (Fig. S1) was constructed using the online tool Phylomatic (http://www.phylodiversity.net/phylomatic/phylomatic.html). Phylogenetic signal intensity of all leaf traits (functional traits and chemical traits) were measured by K statistics^49^ to detect if a given trait of species is phylogenetically conserved. Phylogenetic independent contrasts (PIC) was employed to remove the phylogenetic relatedness of the pairwise correlations among leaf traits^50^. The phylogenetic analysis was performed in “picante” package^51^.

All statistical analyses were performed using R software, version 3.2.1 (R Development Core Team 2011).

### Data availability

The datasets generated during and/or analyzed during the current study are available from the corresponding author on reasonable request.

## Electronic supplementary material

Supplementary meterial
