# Supplementary material for: Nutrient resorption or accumulation of desert plants with contrasting sodium regulation strategies
Source: Sci Rep. 2017 Dec 6;7:17035. doi: 10.1038/s41598-017-17368-0 (PMC5719051; doi:10.1038/s41598-017-17368-0)
Supplement: Supplementary file 1 — Supplementary meterial [file 41598_2017_17368_MOESM1_ESM.pdf]

Nutrient resorption or accumulation of desert plants with contrasting sodium regulation strategies

Lilong Wang[1]. Liang Wang[2]. Wenliang He[1]. Lizhe An[1]. Shijian Xu[1*]

[1]MOE Key Laboratory of Cell Activities and Stress Adaptations, School of Life Sciences, Lanzhou University, Lanzhou 730000, China.

[2] Administration of Anxi Extra-arid Desert National Nature Reserve, Jiuquan, Gansu 736100, China.

[*]Corresponding author: Shijian Xu

Address: No. 222, Southern Tianshui Road, Lanzhou 730000, China.

E-mail address: xushijian@lzu.edu

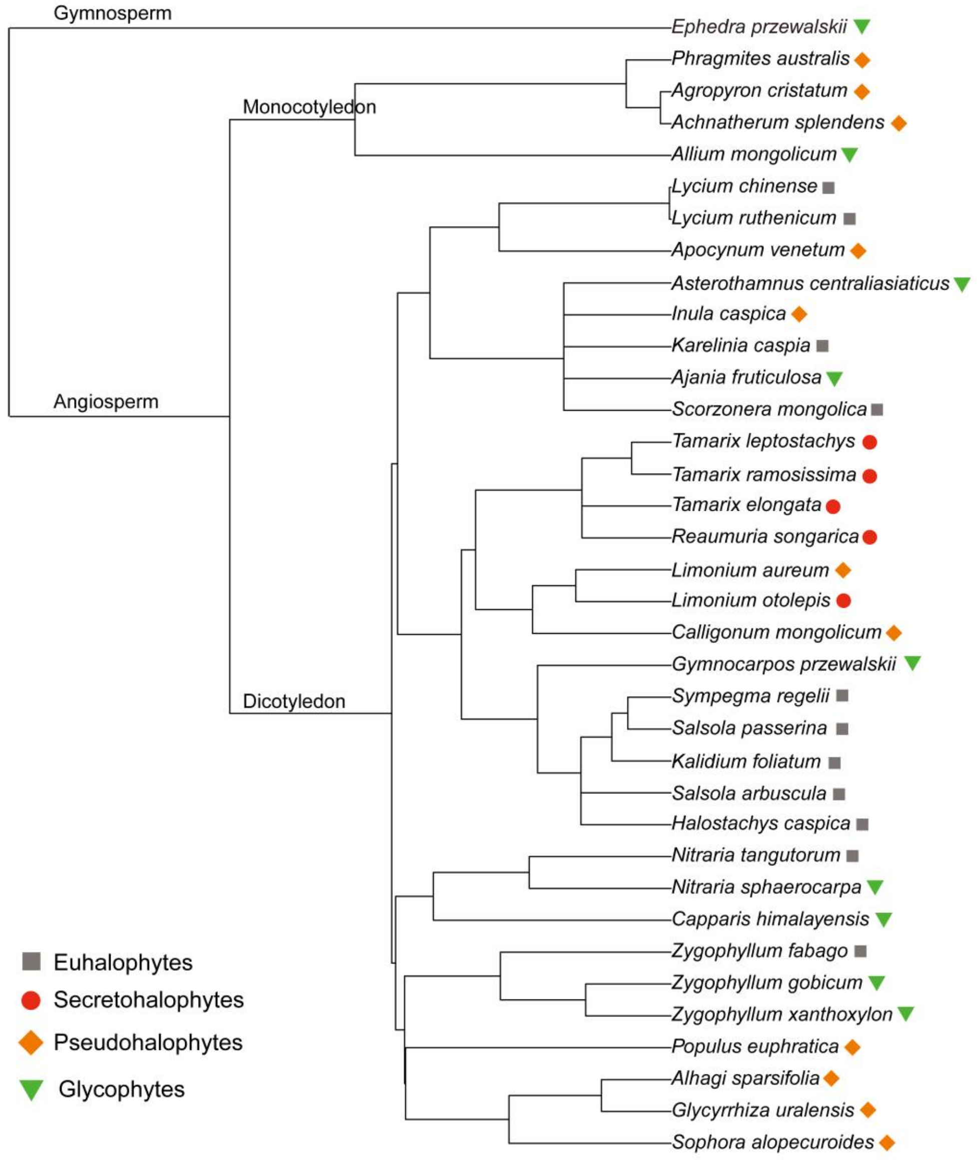

Figure S1. Phylogenetic structure of 36 species in the study area. The phylogeny is based on the Angiosperm Phylogeny Group III classification.

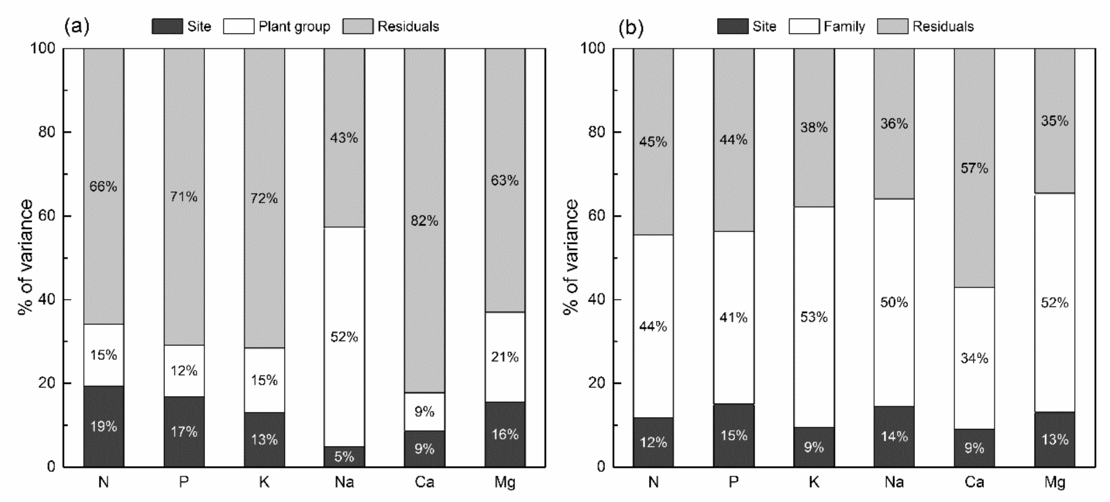

Figure S2. General linear model (GLM) analysis for partitioning the variance of green leaf chemical traits to soil and plant group. (a) site + plant group + residual; (b) site + family + residuals.

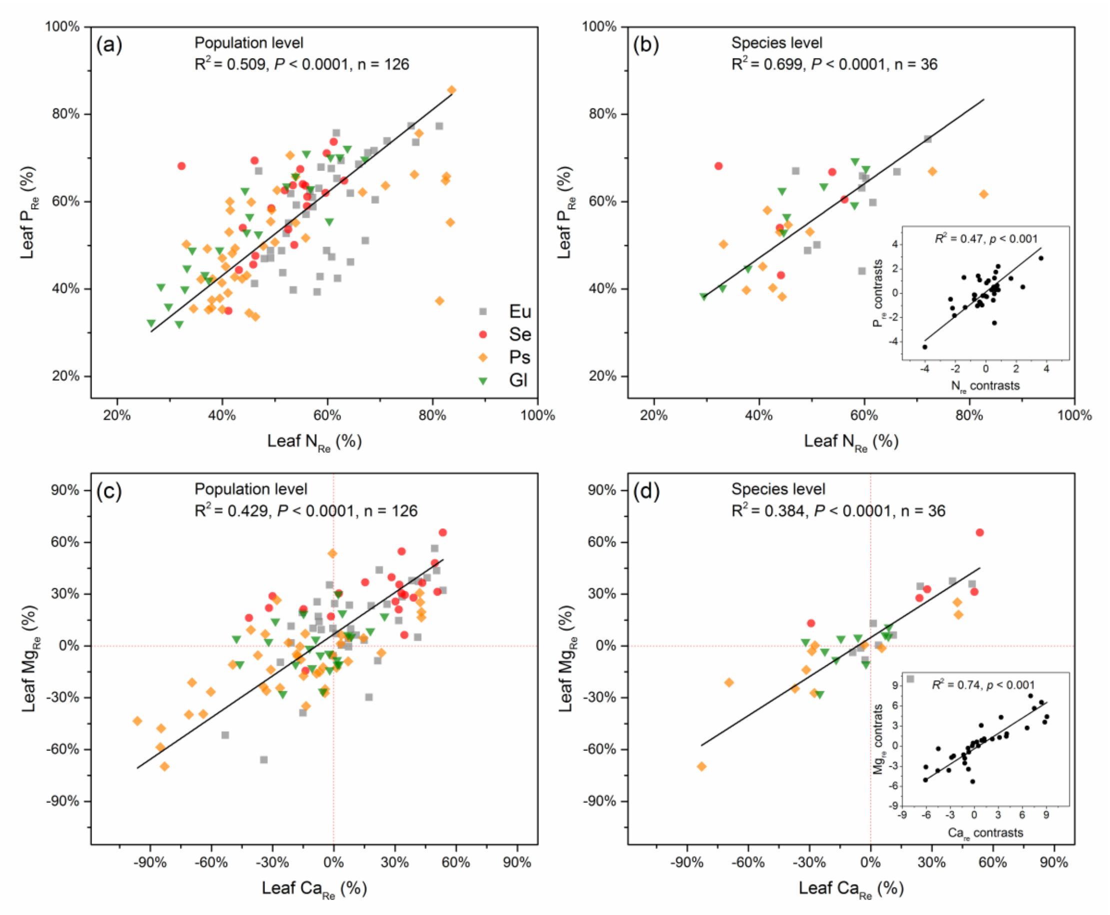

Figure S3. Relationship between nitrogen resorption efficiency (N_{Re}) and phosphorus resorption efficiency (P_{Re}) at population level (a) and species level (b). Relationship between calcium resorption efficiency (Ca_{Re}) and magnesium resorption efficiency (Mg_{Re}) at population level (c) and species level (d). The red dashed line represent 0% resorption. The insets show phylogenetic independent contrast (PIC) correlation at species level.

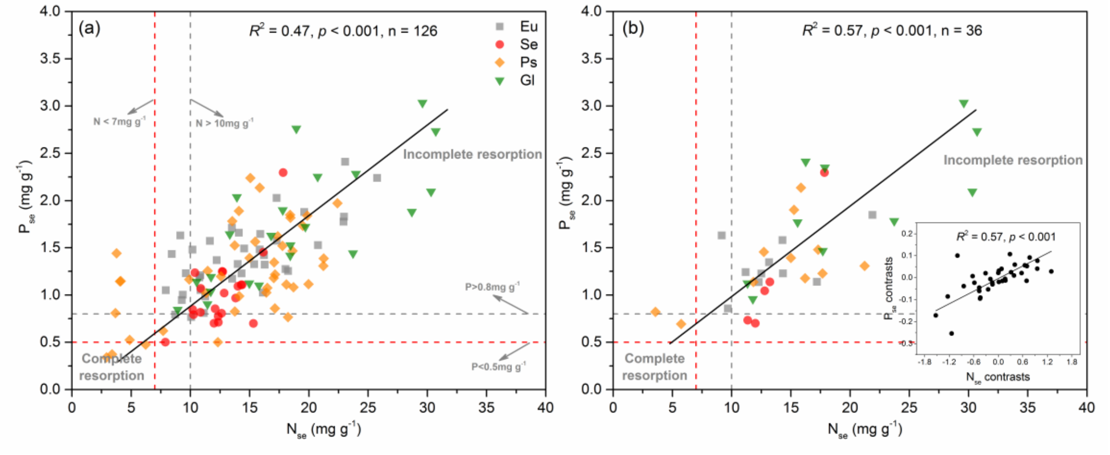

Figure S4. Relationship between nitrogen (N_{se}) and phosphorus (P_{se}) concentration in senesced leaf at population level (a) and species level (b) respectively. The red dashed line represent threshold of complete resorption and the gray dashed line represent threshold of incomplete resorption (Killingbeck 1996). The inset shows the phylogenetic independent contrast (PIC) correlation at species level.

Table S1 Summary of soil and vegetation characteristics in gravel desert and saline area.

Soil & Vegetation characteristics		Habitat	
		Saline area	Gravel desert
Soil properties	Soil EC (ms cm^{-1})	30.5 ±4.30	0.69 ±1.08
0-20 cm	Soil WC (%)	14.7 ±6.05	2.31 ±1.89
	Soil N (mg g^{-1})	0.80 ±0.33	0.22 ±0.07
	Soil P (mg g^{-1})	0.70 ±0.18	0.72 ±0.17
Vegetation	Coverage (%)	35.7 ±2.20	13.7 ±1.15
characteristics	Density (plant m^{-2})	8.47 ±0.90	0.65 ±0.09
	Richness (species $10m^{-2}$)	5.50 ±0.50	3.80 ±0.40
	Dominant species	*Alhagi sparsifolia*	*Allium mongolicum*
		Apocynum venetum	*Ephedra przewalskii*
		Lycium ruthenicum	*Gymnocarpos przewalskii*
		Tamarix leptostachys	*Nitraria sphaerocarpa*
		Tamarix ramosissima	*Zygophyllum xanthoxylon*

Data are Mean±SD (standard deviation). Soil EC (electrical conductivity) was measured in soil: water = 1:5 (weight/volume). Soil WC (water content) measured by (Fresh soil weight – Dry soil weight)/Dry soil weight. Soil N and P represent concentration of total soil N and P. The detail information of dominant species listed in table S3.

Table S2 The factor loading for the six elements on the three principal components (PCA) axes for green and senesced leaves.

	Green leaf			Senesced leaf		
	PC1	PC2	PC3	PC1	PC2	PC3
N	0.25	2.38	-1.21	0.33	-1.12	-0.53
P	0.01	0.34	-0.09	0.04	-0.13	-0.05
K	-1.52	6.61	0.98	0.81	-5.19	0.24
Na	9.97	1.06	-0.29	-12.41	-0.38	-0.07
Ca	0.70	-0.66	1.70	-0.26	0.48	1.49
Mg	1.01	-0.62	3.43	-0.16	-0.02	3.42
Proportion explained	56.5%	28.2%	9.4%	74.5%	13.8%	6.8%

Table S3 List of species sampled in the study area.

Species	Family	Life form	Height (cm)	Group	Abbreviation
Achnatherum splendens	Gramineae	Grass	114.7	Ps	*As.*
Agropyron cristatum	Gramineae	Grass	22.9	Ps	*Ac.*
Ajania fruticulosa	Compositae	Subshrub	34.0	Gl	*Af.*
Alhagi sparsifolia	Leguminosae	Herb	47.1	Ps	*Asp.*
Allium mongolicum	Liliaceae	Herb	11.5	Gl	*Am.*
Apocynum venetum	Apocynaceae	Subshrub	68.3	Ps	*Av.*
Asterothamnus centraliasiaticus	Compositae	Subshrub	24.9	Gl	*Ace.*
Calligonum mongolicum	Polygonaceae	Shrub	105.4	Gl	*Cm.*
Capparis himalayensis	Capparaceae	Subshrub	30.6	Gl	*Ch.*
Ephedra przewalskii	Ephedraceae	Subshrub	16.6	Gl	*Ep.*
Glycyrrhiza uralensis	Leguminosae	Herb	51.5	Ps	*Gu.*
Gymnocarpos przewalskii	Caryophyllaceae	Subshrub	57.9	Gl	*Gp.*
Halostachys caspica	Chenopodiaceae	Shrub	76.9	Eu	*Hc.*
Inula caspica	Compositae	Herb	21.5	Ps	*Ic.*
Kalidium foliatum	Chenopodiaceae	Subshrub	30.9	Eu	*Kf.*
Karelinia caspia	Compositae	Herb	41.9	Eu	*Kc.*
Limonium aureum	Plumbaginaceae	Herb	30.3	Ps	*La.*
Limonium otolepis	Plumbaginaceae	Herb	40.6	Se	*Lo.*
Lycium chinense	Solanaceae	Subshrub	26.7	Eu	*Lc.*
Lycium ruthenicum	Solanaceae	Subshrub	39.1	Eu	*Lr.*
Nitraria sphaerocarpa	Zygophyllaceae	Subshrub	24.7	Gl	*Ns.*
Nitraria tangutorum	Zygophyllaceae	Subshrub	29.4	Eu	*Nt.*
Phragmites australis	Gramineae	Grass	31.8	Ps	*Pa.*
Populus euphratica	Salicaceae	Tree	386.7	Ps	*Pe.*
Reaumuria songarica	Tamaricaceae	Subshrub	30.17	Se	*Rs.*
Salsola arbuscula	Chenopodiaceae	Subshrub	13.8	Eu	*Sa.*
Salsola passerina	Chenopodiaceae	Subshrub	26.8	Eu	*Sp.*
Scorzonera mongolica	Compositae	Herb	3.8	Eu	*Sm.*
Sophora alopecuroides	Leguminosae	Herb	55.7	Ps	*Sal.*
Sympegma regelii	Chenopodiaceae	Subshrub	49.4	Eu	*Sr.*
Tamarix leptostachys	Tamaricaceae	Shrub	139.2	Se	*Tl.*
Tamarix elongata	Tamaricaceae	Shrub	125.9	Se	*Te.*
Tamarix ramosissima	Tamaricaceae	Shrub	128.5	Se	*Tr.*
Zygophyllum xanthoxylon	Zygophyllaceae	Shrub	56.5	Gl	*Zx.*
Zygophyllum fabago	Zygophyllaceae	Herb	20.4	Eu	*Zf.*
Zygophyllum gobicum	Zygophyllaceae	Herb	1.1	Gl	*Zg.*

All species are perennial and winter deciduous. Plant height was averaged by all individuals of each species.

Table S4 Species sampled in each site.

	Site	latitude	longitude	Soil EC (ds/m)	Species sampled
Saline area (S)	S1	40°21′13.22″	95°52′13.52″	15.3	*Asp. Lr. Pa. Sm. Tl.*
	S2	40°14′43.15″	96°05′04.60″	12.7	*Ha. Kf. Nt. Tl. Zf.*
	S3	40°23′56.97″	96°14′45.31″	16.8	*Av. Pa.*
	S4	40°22′42.63″	96°13′03.96″	54.4	*As. Pa. Pe. Tl.*
	S5	40°15′53.45″	96°10′20.59″	11.8	*As. Asp. Lr. Nt. Pa. Tl.*
	S6	40°30′44.76″	94°58′04.62″	5.84	*Asp. Ch. Kf. Lr. Tl. Tr. Zf.*
	S7	40°34′03.42″	95°00′54.11″	23.4	*Asp. Ha. Lr. Tl. Tr.*
	S8	40°32′23.67″	95°03′04.68″	40.0	*Ha. Kf. Lr. Nt. Pa. Tl. Tr.*
	S9	40°30′43.44″	95°06′21.91″	36.6	*Asp. Av. Pa.*
	S10	40°29′51.39″	95°33′19.15″	91.3	*Asp. Av. Gu. Lr. Pa. Pe. Tl.*
	S11	40°26′06.68″	97°17′53.94″	20.4	*Ac. Asp. Av. Gu. Ha. Kf. Pa. Tr.*
	S12	40°25′36.81″	97°15′45.54″	29.9	*Ac. As. Asp. Gu. Ha. Ic. Kc. Lr. Nt. Pa. Sa. Te. Tr.*
	S13	40°24′55.93″	97°13′54.38″	38.0	*Ac. Asp. Av. Kf. Lc. Lo. Nt. Pa. Pe. Tl.*
Gravel desert (G)	G1	40°03′54.32″	96°16′55.60″	0.19	*Ace. Ep. Gp. Ns. Sp. Sr. Zx.*
	G2	40°04′57.48″	96°11′27.77″	2.63	*Asp. Kf. Ns. Nt. Rs.*
	G3	40°04′45.23″	96°09′52.60″	0.39	*Asp. Cm. Gp. Kf. Lr. Ns. Nt. Pe. Rs. Sr. Zx.*
	G4	40°01′53.96″	96°43′22.19″	0.10	*Ace. Af. Am. Ep. Gp. Ns. Rs. Sar. Sp. Zg. Zx.*
	G5	40°12′32.32″	96°57′43.14″	0.16	*Ace. Cm. Ep. Gp. La. Rs. Sar. Sr. Tr. Zg.*

Soil EC, soil electrical conductivity, 0-20cm. Species names are listed as abbreviation, see Table S3 for the corresponding full name.
